# Mindfulness-based stress reduction group training improves of sleep quality in postmenopausal women

**DOI:** 10.1186/s12888-022-03869-4

**Published:** 2022-04-11

**Authors:** Samaneh Darehzereshki, Fahimeh Dehghani, Behnaz Enjezab

**Affiliations:** 1 Counseling in Midwifery, Faculty of Nursing and Midwifery, Shahid Sadoughi University of Medical Sciences, Yazd, Iran; 2 Department of Psychology, Faculty of Psychology and Educational Sciences, Yazd University, Yazd, Iran; 3 Department of Midwifery, Research Center for Nursing and Midwifery Care, Shahid Sadoughi University of Medical Sciences, Yazd, Iran

**Keywords:** Mindfulness, Sleep Quality, Postmenopausal women, Counseling, Group training, Pittsburgh questionnaire

## Abstract

**Background and purpose:**

Sleep disorder is one of the most common problems during menopause, which affects different areas of people's lives. Today, mindfulness is one of the concepts that have attracted a lot of attention due to its desirable effects and low side effects. The present study was conducted to investigate the effect of mindfulness-based stress reduction (MBSR) group training on sleep quality of postmenopausal women.

**Method:**

This is a quasi-experimental parallel study. The study involved 66 eligible postmenopausal women 45–60 years’ old (*n* = 33 in each group) during 2019–2020. The minimum score of women was 5 from Pittsburgh questionnaire. Eight sessions weekly (120-min/session) MBSR group training was conducted in the intervention group and menopausal health in the control group for two months. Pittsburgh Sleep Quality Questionnaire was used as a data collection tool. Descriptive statistics and nonparametric tests were utilized to analyze the data using SPSS software (version 25). The significance level < 0.05 was considered.

**Results:**

Socio-demographic characteristics of participants were no statistically significant difference between the study groups before the intervention. The results showed that mean of overall quality of sleep score was before the intervention 10.21 ± 3.03, after the intervention 4.7 ± 2.45, and one month after the intervention 4.69 ± 2.4 respectively in intervention group (*P* < 0.001). While there was no significant change in the mean overall quality of sleep quality in the control group.

**Conclusion:**

According to the results, MBSR group training is an effective strategy for improving the sleep quality of postmenopausal women. Therefore, could be used to improve the sleep quality of postmenopausal women by midwifery consultants in health centers.

## Background

During recent decades, due to the advancement of medical science and the improvement of life expectancy across the world, numerous women are experiencing menopause [1]. Was estimated that this population will reach 1.2 billion people in 2030 with an annual increase of 47 million new cases [2]. Also, according to statistics provided by the Iran Ministry of Health, about 5 million menopausal women will live in Iran until 2021 [3].

During menopause, women experience changes such as hot flashes, night sweats, tachycardia, headaches, dizziness, fatigue, and irritability due to low levels of hormones. Sleep disorders are one of the most common problems among these women. The range of these symptoms is from mild to severe and debilitating [4, 5]. The prevalence of insomnia increases from 38% in premenopausal women to 46–48% in postmenopausal women [6].

Insomnia has many negative effects on people's quality of life and reduces daily physical, psychological and social performance [7]. Research has revealed that insomnia decreases the body's immune system performance, hypothalamus performance, and mental activities (reduced vigilance, concentration and memory capacity [8]) and increases the risk of heart attacks [9, 10]. Strine et al. study (2005) revealed that changes in the secretion of female hormones, stress, disease, lifestyle, and sleep environment are effective in the development of sleep disorders [11]. Several factors can cause sleep disorders including, biological, cognitive, and behavioral factors [12].

Non-pharmacological interventions, especially behavioral interventions due to their low side effects, have found great popularity for improving sleep disorders [13]. Often, people who have trouble falling asleep have more emotional and stressful states compared to others. In particular, pre-sleep thoughts play an important role in insomnia [14]. These people have a higher level of arousal and anxiety and are more affected by life stressors [14, 15].Treatment of insomnia have approved with Interventions that focus on reducing stress and relieving anxious and disturbing thoughts [16].

Mindfulness is one of the concepts that have recently attracted a lot of attention. Mindfulness means being aware of thoughts, actions, emotions, and feelings and is a special form of attention [17]. In other words, mindfulness means being in the moment without judging or commenting on what will happen. That is the experience of pure reality without any explanation or interpretation [18]. Mindfulness is a way to live a better life, relieve pain, to enrich life and to make life meaningful [19]. In a person with sleep disturbances, persistent rumination about to fall asleep during the day or night, causing physical and emotional arousal in her/his [20]. Mindfulness training had positive effects on pain, attention, and sleep problems in patients with chronic pain [21]. The most common method of mindfulness training is mindfulness based stress reduction (MBSR), which is known as a stress reduction program and relaxation training program [22]. MBSR training included: 1) body-scan exercises, 2) mental exercises focusing one's attention on the breath, 3) physical exercises with focus on being aware of bodily sensations and one's own limits during the exercises, and 4) practicing being fully aware during everyday activities by using the breath as an anchor for the attention [23].

Using MBSR as a treatment for insomnia in cancer patients can increase the overall sleep time, reduce stress levels, and reduce mood disorders [24]. Research reveals that mindfulness training improves sleep quality [25] and sleep parameters [26] of women with breast cancer, physical symptoms of women with irritable bowel syndrome [27], hot flashes [28], menopausal symptoms [29], and quality of life in perimenopausal women [30], depression and anxiety [31], increases mental well-being and hope of patients with multiple sclerosis (MS) [32], improves protective factors against sleep, anxiety, depression, and fatigue in patients with MS [33], and decreases perceived pain intensity and performance limitation of women with chronic pain [34].

Due to the prevalence of sleep disorders in postmenopausal women, the harmful effects of it’s on human health, also the lack of studies on the effectiveness of midwifery counseling services for postmenopausal women with sleep disorders, and limited studies on this field in Iran, the present study was performed to investigate the effect of mindfulness-based stress reduction group training on sleep quality of postmenopausal women in Yazd-Iran.

## Materials and methods

### Study design and participants

The present study was a quasi-experimental, parallel, and three-stage design (pre-test, post-test, follow-up test) and had an active control group. In the first, was obtained the ethics code (IR.SSU.REC.1397.181) and necessary introduction letters. The study population consisted of all eligible postmenopausal women in one of the Comprehensive Health Centers in Yazd-Iran. According to the study of David Black et al. [35], and taking into account the confidence level of 95%, power of 80%, the mean of difference scores of sleep quality of 1.3, and standard deviation of sleep quality score of 1.5 and 2 respectively for the intervention and control groups, and a drop of 20% in each group, the sample size was considered to be 33 persons in each group. The inclusion criteria were postmenopausal educated Iranian women aged 45–60 years (at least one year since their last normal menstruation without hysterectomy or oophorectomy) and diagnosed with sleep disorders according to a score of at least 5 out of the sleep quality questionnaire. The exclusion criteria were restless legs syndrome, physical and mental illnesses requiring hypnotic drugs, consuming tobacco, alcohol and herbal medicine, hormone therapy, major stressors in the last six months, such as loss of family and divorce, receiving a psychological intervention or counseling such as mindfulness in the past or at the current time. Also, the absence of at least two sessions, major stressors and severe stressful events in each stage of the plan, missing at least 70% of the homework during the study, the presence of physical or mental illness that required the use of sleeping pills or created a sleep disorder during the study were criteria of drop.

### Sampling& randomization

The researcher referred to the health centers of Yazd-Iran for sampling. Sampling was according to the inclusion and exclusion criteria, which lasted two months. Using Health system database of Yazd province, a list of eligible postmenopausal women ages (45 to 60 years) with their phone numbers were obtained. During the telephone call, the objectives of the study were explained to individuals. Eligible volunteers were invited to a face-to-face meeting and completing the demographic questionnaire at the beginning of the session and were re-evaluated for other inclusion and exclusion criteria (Completing the Pittsburgh Questionnaire (PSQI) and get a score above 5).

**Fig. 1:**
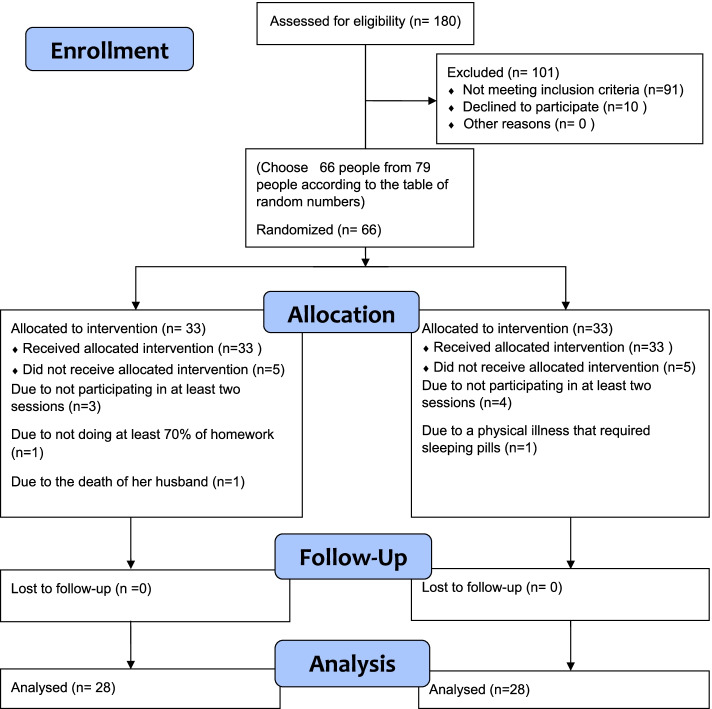
Participant’s flow diagram

Finally, out of 79 postmenopausal women, 66 persons randomly were selected by a random number table. Then 66 postmenopausal women were randomly assigned into two groups: control (*n* = 33) and intervention groups (*n* = 33) using the table of random numbers (Fig. 1). All participants were informed about the study and completed a written informed consent before participating in the study. The participants of both groups completed the questionnaires before the intervention as a pre-test, immediately after and one month after the intervention. The sessions were conducted under the supervision of a PhD in Psychology and by a person trained in mindfulness courses.

### Data collection tools

To collect data, the socio-demographic questionnaire and Pittsburgh Sleep Quality Questionnaire was used in this study.

### Demographic questionnaire

Demographic questionnaire included personal information such as: age, level of education, occupation status, and duration of menopause that was completed at the first meeting.

### PSQI

The Pittsburgh Sleep Quality Questionnaire (1989) basically has 9 items, but the fifth question contains 10 sub-items and items are scored based on a 4-point Likert scale from 0 to 3. This questionnaire has 7 subscales which include: sleep efficiency, subjective sleep quality, sleep latency, sleep duration, Sleep disturbances, day time dysfunction, and overall sleep quality with an overall scoring range of 0–21. After the final scoring, a score of 5 or more indicates sleep disorder. The higher score indicates the worse quality of sleep. Numerous studies support the validity and reliability of this questionnaire [36]. Buysse et al. (1989), the developers of the scale, estimated the reliability of this scale to be 0.83. Burkhulter et al. obtained the reliability of 0.83 using Cronbach's alpha coefficient [37]. Farrahi Moghaddam et al. assessed the reliability and validity of the Persian version of the Pittsburgh Sleep Quality Index (PSQI-P) and Cronbach's alpha coefficient for all subjects was 0.77 [38]. Asadnia et al. also reported reliability of the Persian version of this questionnaire 0.82 using Cronbach's alpha coefficient [39].

### Intervention

The framework of mindfulness sessions was set on Mindfulness-Based Stress Reduction (MBSR) Authorized Curriculum Guide [40], Mindfulness book [41], and Rochelle L. Adams books [42]. The content of menopausal health training on Novak's book [43] and “Management of the Menopause” books [44] (Tables 1 and 2), and were held in 8 sessions of 120 min weekly for two groups from December2019 to March 2020.To prevention of communication between the two groups, the meetings of the intervention group were held in the Aliebn Abitaleb Mosque and the control group in the Qamar Bani Hashem Mosque. Then, they have presented their homework for each session. After this, the participants of both groups completed the Pittsburgh questionnaire immediately after and one month after the intervention. During the one-month follow-up period, due to the onset of the Coronavirus outbreak, the questionnaires were completed by telephone.

**Table 1:** Content of the intervention sessions for the intervention group

Meeting	Content of the meeting	Homework
First	-pretest - setting goals of the sessions - Teaching the concepts of MBSR, eating raisin practice - physical examination Practice - focusing on short breathing - completion of the class with Focus on short breathing 2 to 3 minutes of breathing) - Determining homework	Doing one of your daily activities such as tooth brushing and recording it in the homework book
Second	- body scan- physical examination Practice - Practice review – Homework review - thoughts and feelings Practice (walking in the street) - Recording pleasant and enjoyable events - Sitting meditation for 10 to 15 minutes - Determining homework	Mindful Breathing for 10–15 minutes, 6 times in 7 days. –body scan- Recording pleasant or enjoyable events every day - mindfulness in normal life activities
Third	- seeing or hearing Practice for 50 minutes - Sitting meditation for 30 to 40 minutes - Review Practice 3- minute breathing space Practice and reviewing it - Preparing a list of unpleasant events	- Preparing a daily list of unpleasant events - Have a breathing space three times a day - Sitting meditation for 30 to 40 minutes a day and recording it in the homework book
Fourth	- 5 minute practice of seeing or hearing - 40 minutes of meditation - Awareness of breathing, body, sounds and thoughts - 3- minute breathing space and reviewing it.	-3 minute breathing space regularly three times a day -3 minute breathing space - coping in case of unpleasant feelings caused by menopausal complications) - 40 minutes of meditation per day and recording it in the homework book
Fifth	- Forty minutes of sitting meditation - Awareness of breathing, body, sounds, thoughts. Paying attention to reactions to thoughts, feelings, or bodily sensations. Expressing the difficulties that occur during practice. Their effects on the body and reaction to them.	-3 minute breathing space - coping in case of unpleasant feelings caused by menopausal complications– 40 minutes of sitting meditation per day and recording it in the homework book
Sixth	- 40 minute sitting meditation. Awareness of breathing, body, sounds and thoughts in addition to paying attention to reactions to problems ) - Creating, thinking and practicing point of view or surrogate thoughts	practicing at least 40 minutes a day -three minutes breathing space - coping when faced with unpleasant side effects of menopause and recording it in a homework book
Seventh	-40 minutes sitting meditation - Awareness of breathing, body, sounds, thoughts (in addition to paying attention to reactions to problems) - Observing the relationship between activity and mood - Preparing a list of enjoyable activities that lead to a sense of accomplishment. - 3- minute breathing space as the first step to have mindfulness. - 3- minute breathing space or mindful walking.	Choose a pattern from different ways, for the practice you intend to use systematically– 3 minutes breathing space– Regularly for 3 times a day - 3 minutes of breathing space - coping when faced with the unpleasant effects of menopause and recording it in the homework book
Eighth	Physical examination practices - Homework review - Reviewing the entire program. - Discussing how to continue the mobility and discipline that has developed in the last 7 weeks in regular practices - Checking and discussing programs and finding positive reasons to continue practice - post-testing

**Table 2:** Content of the intervention sessions for the control group

Meeting	Issue	Content of the meeting
First	Osteoporosis	Definition of the disease and its severity—Ways of prevention from osteoporosis—Risk factors
Second	Osteoporosis	Osteoporosis Diagnosis—Osteoporosis side effects- Osteoporosis treatment
Third	Cardiovascular disease	Definition of the disease and its severity—Ways to prevent cardiovascular disease—Risk factors
Fourth	Cardiovascular disease	Effects of menopause on cardiovascular disease Effects of hormone therapy on cardiovascular disease
Fifth	Cervical cancer and abnormal bleeding	Definition of the disease and its severity—Ways of prevention from cervical cancer disease—Risk factors
Sixth	Cervical cancer and abnormal bleeding	Cervical cancer diagnosis and screening—Treatment—The importance of bleeding
Seventh	Breast cancer screening	Definition of the disease and its severity—Ways of prevention from breast cancer disease—Risk factors
Eighth	Breast cancer screening	Diagnosis-Breast cancer screening—Treatment and the post-test

### Data analysis

Data were analyzed by SPSS software version 25. Descriptive statistics wereused to describe demographic variables including mean and rankings mean and inferential statistics were used to analyze and find connections. Fisher's test and Independent t-test were used to compare socio-demographic. The normality of variables was examined using the Kolmogorov–Smirnov test, and due to the lack of normal distribution, nonparametric tests (Friedman and Mann–Whitney U test) and Bonferroni post hoc test were used. *P* < 0.05 was considered for the confirmation or rejection of hypotheses.

## Results

Among the 180 women aged 45 to 60, 79 were eligible to take part in this study. 66 women were chosen randomly in the study, and 66 women were allocated randomly to the intervention [33] and active control groups [33]. They participated in 8 intervention sessions. A number of participants dropped due to not to participating in at least two sessions, failure to do homework, major stressor, and need for sleeping pills (intervention group = 28 and control group = 28) (Fig. 1).

The results showed the mean age of postmenopausal women participating in the study was 53.2 ± 4.2 years, and the mean duration of post menopause was 5.5 ± 3.9 years. The majorities of participants had high school education and were housewives. The demographic characteristics of the subjects in the intervention and control groups are not significantly different from each other (Table 3) (*P* > 0.05).

**Table 3:** Socio-demographic characteristics in the study groups

Variable		Intervention group	Control group	Total	*p*- Value
Occupational status		No(Frequency)	No(Frequency)	No(Frequency)	
	Housewife	27(96.42)	27(96.42)	54(96.42)	> 0.05*
	Employed	1(3.57)	1(3.57)	2(3.57)	> 0.05*
Education level					
	High school	26(92.58)	22(78.56)	48(85.71)	0.2*
	Diploma and higher	2(7.14)	6(21.42)	8(14.28)	0.2*
		Mean ± SD	Mean ± SD	Mean ± SD	*p*- Value
Age		52.9 ± 4.4	53.6 ± 4.1	53.2 ± 4.2	0.5**
Duration of post menopause		5.08 ± 3.2	6.03 ± 4.6	5.5 ± 3.9	0.3**

^*^Fisher's test

^**^Independent t-test

The component of the use of sleep medications has not been studied because one of the inclusion criteria was not using sleep medications. Therefore, among the 6 components of sleep quality that were studied; participation in the MBSR course had an effect on the components of sleep efficiency, subjective sleep quality, sleep latency, sleep duration, day time dysfunction, and overall sleep quality score immediately and one month after the study. There is a significant difference between the intervention and control groups (*p* < 0.05). Mindfulness did not affect the sleep disturbances component immediately after the study (*p* = 0.31) but affected the one-month follow-up period (Table 4) (*p* = 0.01).

**Table 4:** Comparison of sleep quality and its component between study groups

sleep quality	Intervention group	control group	*p*-value *
**Sleep efficiency**			
Before intervention	1.28 ± 1.04	1.67 ± 1.02	-
Immediately after the intervention	0.57 ± 0.38	1.53 ± 1.07	0.001
One month after the intervention	0.58 ± 0.28	1.67 ± 0.98	< 0.001
*p*-value **	< 0.001	0.17	
**Subjective sleep quality**			
Before intervention	1.57 ± 0.74	1.39 ± 0.56	-
Immediately after the intervention	0.82 ± 0.54	1.3 ± 0.55	0.001
One month after the intervention	0.81 ± 0.56	1.28 ± 0.46	0.008
*p*-value **	< 0.001	0.27	
**Sleep latency**			
Before intervention	2.32 ± 0.72	1.92 ± 0.6	-
Immediately after the intervention	0.96 ± 0.69	1.85 ± 0.44	< 0.001
One month after the intervention	1 ± 0.72	1.82 ± 0.54	< 0.001
*p*-value **	< 0.001	0.55	
**Sleep duration**			
Before the intervention	1.85 ± 0.89	2.14 ± 0.59	-
Immediately after the intervention	1 ± 0.81	2.14 ± 0.63	< 0.001
One month after the intervention	0.96 ± 0.79	2.03 ± 0.65	< 0.001
*p*-value **	< 0.001	0.16	
**Sleep disturbances**			
Before intervention	1.57 ± 0.5	1.25 ± 0.44	1.25 ± 0.44
Immediately after the intervention	1.14 ± 0.35	1.25 ± 0.44	1.25 ± 0.44
One month after the intervention	1 ± 0.27	1.25 ± 0.44	1.25 ± 0.44
*p*-value **	< 0.001	> 0.05	> 0.05
**Day time dysfunction**			
Before intervention	1.07 ± 0.71	0.64 ± 0.48	-
Immediately after the intervention	0.28 ± 0.46	0.63 ± 0.47	0.008
One month after the intervention	0.27 ± 0.45	0.57 ± 0.5	0.03
*p*-value **	< 0.001	> 0.05	
**Overall sleep quality score**			
Before intervention	10.21 ± 3.03	9.03 ± 2.2	-
Immediately after the intervention	4.7 ± 2.45	8.7 ± 2.19	< 0.001
One month after the intervention	4.69 ± 2.4	8.7 ± 1.8	< 0.001
*p*-value **	< 0.001	0.35	

*P*-value *: Mann–Whitney test for comparison between two groups

*P*-value **: Friedman test for comparison within each group

In the present study, participants did not report any adverse effects related to participating in the mindfulness training course.

According to Bonferroni post hoc test was statistically significant differences between the total score of sleep quality of women before and immediately after intervention, also before and one month after the intervention. But were not statistically significant differences between immediately after intervention with one month after the intervention (Table 5).

**Table 5:** Results of Bonferroni post hoc test for sleep quality

Comparison of intervention group	Mean difference	standard error	*P*- value
Before the intervention—immediately after the intervention	1.41	0.26	< 0.001
Before the intervention—one month after the intervention	1.48	0.26	< 0.001
One month after the intervention—immediately after the intervention	0.071	0.26	0.8

## Discussion

This study revealed that MBSR group training affects the sleep quality of postmenopausal women until one month after the intervention (*p* < 0.001). The results of the present study are consistent with some studies [35, 45–48]. Andersen et al.(2013) assessed effect of mindfulness-based stress reduction on sleep quality among Danish breast cancer patients; their results showed that MBSR improved of sleep quality just after the intervention but no long- term effect (6–12 months) among these patients [25]. Also in Andersen's study, it is recommended to study people with sleep disorders, which have been considered in this study.

According to the results of Y. Zhao et al.(2020), mindfulness had a positive effect on the sleep quality of the breast cancer survivors with insomnia [49]. Also D. Zhao et al. (2019) indicated that mindfulness may play a mediating role between neuroticism and sleep quality in people with asthma. High neuroticism is associated with low levels of mindfulness, that has correlated with poor sleep quality and vice versa. In neurotic patients, there is a greater tendency to pay attention to negative emotions, and performing mindfulness interventions can with reducing neuroticism improved the quality of sleep [50]. MBSR is effective on the sleep quality, depression, anxiety, and stress in people with type 2 diabetes compared to the control group. MBSR by more acceptance of unchanging life events provides a new way to deal with stress [51].

Talley et al. (2020) in a comprehensive study revealed that a high level of intrusive thoughts, avoidance, and over-stimulation are associated with sleep quality. Mindfulness techniques due to improving awareness and reducing reaction to negative thoughts can help a person to sleep [48]. Of course, in the present study, the mediators of sleep quality have not been investigated. Also researches demonstrate that consciousness is associated with reduced sleep disorders and improved sleep quality through awareness [52–54].

MBSR is an intervention that allows a nonreactive awareness to an experience. For instance, some women may react to a hot flush with a sense of despair and concern that the hot flushes will never stop and that they experience sleep disorders. The MBSR arm significantly improved in hot flushes, quality of life, sleep quality, anxiety, and perceived stress [55].

Also, the results of Farahbakhsh et al. (2016) revealed that ​mindfulness treatment affects sleep quality and mental health of patients with insomnia [20]. Which is consistent with the results of the present study, but the effects during follow-up and the degree of stability of the effects has not been measured in the mentioned study.

The results of KazemiZahrani and Behnampour (2020) revealed that mindfulness-based stress reduction therapy had positive effects on anxiety and sleep quality [46] That is consistent with the results of the present study. Their findings indicate that mindfulness affects the components of sleep delay, sleep duration, and daily dysfunction, which is consistent with the present study and has not to effect on the components of sleep quality, sleep disorders, and useful sleep, which compared to this study only, is consistent with sleep disorders. In other studies, MBSR for sleep quality in short-term follow-up (1–3 months) was effective, whereas long-term follow-up (6–12 months) was associated with decreased efficacy [25, 45].

On the other hand, the results of the present study are not consistent with the study of Van der zwan et al. and Shapiroetal. Van der zwan et al. (2015) compared the effectiveness of three methods in reducing stress and related symptoms. Their results revealed a generally beneficial effect, including reducing stress, anxiety and depressive symptoms and improving psychological well-being, but the interventions did not have a statistically significant effect on sleep quality [56]. Also, a study by Shapiro et al. revealed that both the MBSR group and the control group had significant improvements in daily sleep quality, although none of them had a significant improvement in sleep performance [57].

### Strengths and limitations

One of the strengths of this study was having active control, and follow-up period. Also, participants were entered in this study with sleep disorders (the score of at least 5 out of the sleep quality questionnaire). Synchronicity with the corona virus pandemic was one limitation of this study of the follow-up period. Therefore, the questionnaires in follow-up period were completed by telephone. Also follow-up period was the one month that cannot represent a lasting effect. One of the limitations this study was that is not examined the role of mediators such as negative stimulations, emotion regulation, perceived stress, and etc. It is suggested that the role of mediators be examined in future studies.

## Conclusion

Based on the results of this study, MBSR group training caused improvement in sleep quality in postmenopausal women. Therefore, its use is recommended as a non-pharmacological method to prevent and treat this common problem in postmenopausal women. Also, since sleep quality is a basic dimension of quality of life and, improving the quality of life of postmenopausal women is one of the important goals of women's health. As a result, with proper training to health care providers in the fields such as MBSR, they can promote the quality of life of postmenopausal women.

## Data Availability

The data and materials are available on request from the corresponding author (BE).
